# Book Review

**Published:** 1938

**Authors:** 


### Reviews of Books

**A Cerebral Atlas.** By R. J. A. Berry, M.D., F.R.S.E., F.R.C.S. Pp. xxvi., 425. Illustrated. London: Oxford University Press (Humphrey Milford). 1938. Price £5 5s. 0d.—Haeckel's dictum "that every psychic action requires the complete and normal condition of the correlative brain structures for its full and normal exercise" contains a basic truth which sometimes is overlooked in the study of mental deficiency. Professor Berry, the Director of Medical Services at Stoke Park Colony, is one of the pioneer workers who has based his researches on this sure foundation. One of his earliest papers, published in 1913, in the *Proceedings of the Royal Society of Victoria*, dealt with the cubic capacity of the brain in 355 criminals. This investigation was undertaken to discover whether there exists a physiological correlation between the size of the head and intelligence. During the quarter-century that has elapsed, Professor Berry has had unusual opportunities, first in Australia and then in England, for a continuous study of mental deficiency. His conclusion is unshaken that what is termed "mind" is a function of the "brain," and that the development of a normal human intellect demands the development of a normal human brain. The *Cerebral Atlas*, now under review, consists primarily of a photographic comparison of the brains of 120 mental defectives with the brains of 77 reputedly normal individuals. The mental defectives' brains were unselected, and represent 120 consecutive necropsies. They are, in this *Atlas*, compared age for age with the normal brains. Not only, however, are the naked-eye features of the brains compared, but as the mental and other reactions of the defective individuals were known and observed during life, it has been possible to give an account of the physical, neurological, and mental phenomena and to correlate these findings with the brain after death. The photographs which are included in the *Atlas* demonstrate most convincingly the differences in size between normal and defective brains. Briefly expressed, the defective brain is seen to differ from the normal in being lighter, smaller, and having a smaller cortical area, whilst in many instances the defective retains in its adult condition features of the embryonic or fœtal brain. Professor Berry brings this last point into prominence by giving on page xxi a table comparing the retention of embryonic or fœtal features in defective and normal brains. Thus he emphasizes the importance of studying mental deficiency from the embryological standpoint. The more deeply mental deficiency is studied the clearer it becomes that mental defect is closely allied to cerebral defect, and that cerebral defect is much more frequently of pre-natal than post-natal origin. In his discussion of the numerous developmental errors found in this series of brains, Professor Berry gives a clear and succinct calendar of the intra-uterine development of the fœtal brain. He is able, moreover, to demonstrate the date during the intra-uterine life at which embryological development was arrested or grossly interfered with in many of the brains illustrated. Apart from gross arrest or interference, some consideration is given to cortical histogenesis, and to the histopathology of the defective brain. Eighty per cent. of defective brains show such obvious evidence of aberrant cellular development in the cortex as to be incompatible with normal mental functions : in the remaining 20 per cent. the gross errors of development or pathological departure from the normal are sufficient to explain the intellectual defects. With each set of brain photographs a full description is given of the individual, namely :—

County or Origin. Sex. Age.
Legal classification under the Mental Deficiency Acts.
Mental state during life in one or more different ways.
(*a*) Developmental grade.
(*b*) Merrill-Palmer mental age.
(*c*) Binet I.Q.
(*d*) Porteus I.Q.
Length, breadth and height of head and brain.
Size of brain.
The brain product (as ascertained by multiplying together length, breadth, and height, and taking the three first figures as an index.)
Proportion of brain product to head product.
Weight of right cerebral hemisphere.
Amount in square centimetres of brain areas.
Proportion of pre-central area to total area.
Height and weight of defectives.
Family and social histories of the defectives (when ascertained).
The mental and neurological reactions of the defectives.
Causes of death.

At the end of the volume are a number of extra excellent photomicrographs of brain sections prepared by Dr. R. M. Norman. These examples tend to show that the cellular construction of the cerebral cortex in mental deficiency is as fundamentally erratic as is the naked-eye anatomy of the brain. This *Cerebral Atlas* is one of the most important contributions to the study of mental deficiency that has ever been published. Yet it is not in itself anything more than an invitation to study. Although a great deal of information may be derived from its pages, in the introduction it is stated that the collection of brains at Stoke Park Colony is available to students or investigators, who may, on application, obtain access to all the original specimens. One hemisphere of each brain is mounted as a permanent museum specimen, the other is preserved for present or future examination. Although it is probable that the conclusions which Professor Berry draws from his own study of the specimens are incontestable, every opportunity is offered to other workers to study the specimens anew and draw further and possibly different conclusions. For the most part Professor Berry has been content to record facts with the impartiality of a trained observer. He scarcely permits himself to draw conclusions from his observations, but one conclusion is irresistible, and he commits himself to it. In the series of brains studied in this *Atlas* it is almost impossible to explain their many embryonic and fœtal features by any other cause than a pre-natal one. In this series the possibility of the cerebral defects being due to birth injuries can be ruled out in almost every case. Defects in cerebral development, rather than brain injuries or brain disease, are the usual cause of mental deficiency. In the letterpress of the *Atlas* no attempt is made to discuss the causes which bring about such arrests or errors of development. The author mentions as possible causes differences in the genes, faulty embedding of the fertilized ovum in the uterine mucosa, impoverished states of the maternal blood from a variety of causes—all operative in the earliest stages of the embryo—and not excluding other known or possibly pathological processes acting at a later stage on the fœtus. Taken all in all, Professor Berry has made an outstanding study of the morbid anatomy of mental deficiencies, perhaps the most notable study made hitherto. His work has been made possible by the generosity of the Incorporation of National Institutions for Persons requiring Care and Control, and of the present Warden and co-founder, Mrs. R. G. Burden, who in 1933 established the Burden Mental Research Trust. It is under the auspices of this Trust and largely at its expense that this magnificent *Atlas* has been published. As a splendid example of typography it would be hard to over-praise the volume. The Oxford University Press may well be proud of such a production. The brain photographs are artistically reproduced, and the letterpress is printed in beautiful type on art paper with generous margins. In spite of its size this vast folio volume is attractively readable. Printed in gold on the binding is a figure of the brain drawn by Sir Christopher Wren to illustrate Willis's book in 1664 : an unforgettable collaboration of two Oxford professors. This *Cerebral Atlas* is a book which no neurologist, psychologist or psychiatrist can ignore : it offers an explanation of Feuchtersleben's inconvenient aphorism : "History indeed records cases of partially educated scholars and fanatical poets, but it affords no instance of a wise man who had become an idiot."

**Physiology of the Nervous System.** By J. F. FULTON, M.A., D.Phil., S.B.M.D., Sterling Professor of Physiology, Yale University. Formerly Fellow of Magdalen College, Oxford. Pp. xv., 675. With 94 illustrations. London, New York, Toronto : Oxford University Press (Humphrey Milford). 1938. Price 25s. 0d.—A book on such an attractive subject as the physiology of the nervous system naturally arouses one's liveliest interest, for who would not desire to know something of the natural working of his own brain ? Nor is this interest in any way lessened by noting that Professor Fulton dedicates his work, and a learned one at that, to "Students of Medicine, who must bridge the gap between the concepts of Neurophysiology and the problems of Clinical Neurology." With the imperious nature of the command no one will quarrel. The misfortune is that students of medicine never do bridge the gap, with the inevitable consequence that they mostly remain the technical masters of the signs and symptoms of a trade. Professor Fulton's work has many, very many, exceedingly good points, which it is but fair, generous, and fitting should be pointed out. Only in this way can the reader form his own judgement whether this book is for him, or only for those specialists who profess to have a knowledge, however out of date, of the physiology of the nervous system. It is refreshing to find a physiological account of the nervous system commencing with the "receptors"—that is, with the sense organs. It is more usual to find a few, but only a few, grouped together at the end of the book, under the title "Organs of Special Sense," and even these are subordinated to discussions on what appears to us to be the much less important problem of the muscle-nerve preparations of the frog. No one can ever hope even dimly to understand the functional entity of the nervous system who fails to grasp the vast significance of the stimuli to which the nervous system reacts, the mode in which it does so, the nature of their transmission centrally, and their profound influence in making or marring what in our more grandiloquent moods we are pleased to call our minds. Hence the importance of any work which commences at the beginning, that is, with the mode of entry of stimuli into the human brain. This excellent beginning is followed by chapters on the dorsal spinal roots and the dermatones ; the motor unit ; the synapse and the elementary reflex, and central inhibition. Chapters such as these, and all are good, are again an unusually original commencement to the physiology of the nervous system. Then follow nine chapters on more ordinary lines devoted to the several anatomical parts of the central nervous system from the spinal cord upwards. The remainder of the book gives another nine chapters to the cerebral cortex, one to the basal ganglia, one to the cerebellum—a little unusually placed—and then the final one, to which we have been looking forward all the time—" The Nervous System as a Whole "—one page and a half out of the massive total of 543 ! But an explanation is given that " it has not seemed judicious to widen the scope of the book to include the vast and important literature of physiological psychology." In this chapter the author so admirably describes his own book and its purport that we cannot do better than quote it. " The physiologist tends to reduce this vast organ (the nervous system as a whole) to its ultimate anatomical units, or to such groups of units as are involved in the conventional reflex or in certain levels of functional activity." Along these lines—to our thinking a little archaic—the author gives a most admirable account of himself. Almost every chapter is introduced by a valuable historical note, and is concluded with a summary. As an instance of Fulton's general way of dealing with his problems, and one which will give readers of this review some idea of his methods, may be quoted an extract on a part of the nervous system with which most medical men have some familiarity—the motor cortex. Says Fulton: " Holmes and Page May believed that the pyramidal tract took origin solely from the Betz cells in area 4. . . . More recently it has been established that the giant pyramidal cells originating in area 2 and 5b of the parietal lobe also undergo retrograde changes after spinal lesions, and it is clear that they likewise contribute to the corticospinal system. Schroeder, Minkowski, and von Economo and Koskinas believed that they had found evidence of retrograde cell changes in the larger pyramidal cells of Layer V in area 6 following spinal lesions." Whether the idea conveyed by this brief extract of a well-known part of the cerebral cortex—and the extract is typical of the author's method throughout—is the reader's concept of the physiology of the nervous system is for him to decide. If so, he will find Professor Fulton an admirable and learned guide to, and through, the many experimental studies made within recent years on the nervous system of man and animals. Whilst it is more open to doubt whether it bridges the "gap between neurophysiology and clinical neurology" there can be no doubt that even so learned a work fails to give any idea at all of the relationship between nerve and mind. Fulton admits this in his closing sentence when he says: "The elucidation of mental phenomena, normal and abnormal, still remains the most challenging problem of neurophysiology." The reviewer would go farther and add that physiological books of this nature will never solve the problem, unless, indeed, with the aid of the neuropathologists, whom Professor Fulton seems to ignore. Nevertheless, Professor Fulton's book is an indispensable necessity in the library of every scientific neurologist. A work so constructed demands an extensive bibliography, and the one appended is a stupendous one, including as it does 1,361 references to papers on the nervous system, with, in addition, nineteen closely-printed pages of the names of authors referred to in the text. With such an enormous mass of available information one sometimes wonders when even the physiologists are going to begin to digest it.

**Whitla's Dictionary of Treatment.** Eighth Edition. By R. S. Allison, M.D., M.R.C.P., and C. A. Calvert, F.R.C.S.I. Pp. viii., 1,285. London: Baillière, Tindall & Cox. 1938. Price 30s.—When a book reaches an eighth edition after holding its own for forty-six years it is obvious that it has proved useful. It was a marvellous achievement of Sir William Whitla's to have planned and written a dictionary which should cover the whole general practice of medicine, surgery and midwifery in so small a volume. The marvel is that it remains so small and yet so adequate in spite of the tremendous changes and extensions of the healing art. Perhaps the truth is that there have been more changes than extensions, perhaps the old family doctor of the 1870's had to be ready to treat just as many and just the same ills, injuries and emergencies as his successors of to-day. Certainly he had to depend upon his own resources far more. Transport facilities had not brought hospitals so near to his patients, public authorities offered him no help in their treatment. As one looks through the forty pages of closely-printed index, one does not know which causes the greater wonder—that so much information should be compressed into so few pages or that the average general practitioner should have to know so much and carries his knowledge (so wide and so exact) so lightly. The subjects dealt with are nearly all of common occurrence. This is what gives *Whitla's Dictionary of Treatment* its value and its popularity. There must be few of the conditions whose treatment is described which do not come pretty frequently under observation in any general practice. The new editors and their special contributors may be heartily congratulated on this new edition, the first which has appeared since the lamented death of Sir William Whitla in 1933. The type, paper and indexing are admirable, the subject-matter is up to date.

**Symptoms and Signs in Clinical Medicine.** Second Edition. By E. NOBLE CHAMBERLAIN, M.D., M.Sc., F.R.C.P. Pp. xii., 435. Illustrated. Bristol: John Wright & Sons Ltd. 1938. Price 25s.—It is less than two years since the first edition of this excellent work was reviewed in these pages. As this edition does not markedly differ from its predecessor there does not appear to be room for much further comment. A number of illustrations have been added, the laboratory and scientific sections have all been placed together in two chapters at the end of the book, extra electrocardiograms have been inserted, and other minor improvements been made. The book itself remains a most valuable introduction to medical diagnosis for all students. It is scrupulously accurate, beautifully produced, and interestingly written. Both author and publishers are to be congratulated upon a very fine piece of work.

**Minor Medical Operations.** K. HARRIS, M.D., F.R.C.P., and EDITH HARRIS, M.B., B.S., D.P.H. Pp. x., 198. Illustrated. London: H. K. Lewis & Co. Ltd. 1938. Price 7s. 6d.—This small handbook will fill a much-needed want for the newly qualified and the young house physician. Each operation and, what is more important, the complications and difficulties which may arise are clearly described. In a book of this size, certain omissions are bound to occur, and one feels, perhaps, that less time and space might be given to describing the contents of a linseed poultice, and more to the description of lipiodol injection of the lungs. Apart from these small defects, however, this book will be very helpful to those starting in medical practice.

**Treatment in General Practice.** Articles from the *British Medical Journal.* Vol. I. Second Edition. Pp. x., 259. Price 8s. 6d. Vol. II. Second Edition. Pp. xii., 436. Price 10s. 6d. London: H. K. Lewis & Co. Ltd. 1938.—The captious may say that under cover of the title they have been immersed again in the hospital schools. It is true: for general practice thrives by equipment, knowledge and resource, and turns for renewal where these can be found. The first of these volumes came under notice on its earlier issue, comprising respiratory, infective and cardio-vascular disorders. The second volume proceeds to those of the nervous, alimentary and renal systems, the anæmias, and the rheumatic and metabolic affections. Every section has the impress of its author and the interest of its details. "Head Injuries" for the man with surgical bent and accidents about him: "Diabetes" in all its aspects will find adepts in the younger rather than the older men. Diagrams interpret the skiagraphy of the intestinal tube: every doctor should understand what every patient is asking for—an X-ray. Of new drugs, sulphanilamide has come in with the current revision: amidopyrin is responsible for agranulocytosis: the new mercurials can be studied—in cardiac dropsy under "Congestive Failure": in renal under "Nephrosis." Bath, Buxton and Harrogate cannot put their salubrity into a formula, but tell of iodine and gold. Lumbar puncture may become commoner not only in spinal diagnosis, but also in relieving with venesection, accidents dealt with by Dr. Edwin Bramwell and Sir Farquhar Buzzard. Our complacency should take warning from the memoirs of a Victorian peer, abusing the Court physician: "Who could call *him* in for anything serious? He is not even an average old woman."

**Medical Practice in Residential Schools.** By F. G. HOBSON, D.S.O., D.M., F.R.C.P. Pp. xvi., 284. Illustrated. London: Oxford University Press (Humphrey Milford). 1938. Price 10s. 6d.—This book has been compiled by one who obviously has had much experience in work as a school doctor (although, curiously, he is only described as Physician to the Radcliffe Infirmary), and is planned as a *vade mecum* for school doctors. It contains an immense amount of information upon all subjects likely to come before those in medical charge of schools, with chapters upon organization, training, etc., as well as detailed accounts of all diseases likely to be met with. The author holds decided views, as is only right, but is fair in his presentation of his own ideas. He is not a believer in wholesale tonsillectomy, and quotes numbers of rather unconvincing figures in support of his views. He states, for instance, that in *no circumstances* " (italics his) should tonsils be removed for nasal catarrh which can often be successfully treated by attention to the sinuses alone." There are very good chapters on streptococcal infections with excellent suggestions as to sulphanilamide treatment. Doubtless the next edition will refer to the use of the new chemotherapy in pneumococcal infections. He does not mention whole blood from adults as a protector or as a modifier in measles, and "immune globulin" receives favourable mention only in a footnote. Details of cases with dosage would be helpful, as its value appears to be rather doubtful. This book will be found most valuable as a work of reference. It is well printed, has few but excellent illustrations, copious references and annotations, and is a worthy member of a notable series of medical publications.

**Cardiovascular Disease in General Practice.** By T. East, D.M., F.R.C.P. Pp. x., 206. London: H. K. Lewis & Co., Ltd. 1938. Price 10s. 6d.—This book, which is written by one of the authors of *Recent Advances in Cardiology*, provides the practitioner with a sound and practical description of diseases of the heart and blood vessels. Starting with chapters on the interpretation of symptoms and the physical examination of the heart, the author proceeds to a consideration of the morbid physiology and treatment of cardiac failure. Defects in the coronary circulation are then dealt with and this is followed by a chapter on failure of the peripheral circulation, a particularly valuable contribution. After the various types of heart disease and the arrhythmias have been described, a useful chapter is devoted to diseases of the arteries and veins, and the final chapter contains a most practical section on the diagnosis of the normal heart. Throughout the book the rational management and treatment of the conditions dealt with is described in full detail. The book is excellently produced and can be thoroughly recommended to the practitioner who wishes to revise his knowledge of cardiovascular disease.

**Oxygen and Carbon Dioxide Therapy.** By A. CAMPBELL, M.D., D.Sc., and E. P. POULTON, D.M., F.R.C.P. Second Edition. Pp. xvi., 202. Illustrated. London: Oxford University Press (Humphrey Milford). 1938. Price 12s. 6d.—The first edition of this excellent book was reviewed in our columns in 1935. The present edition contains two addenda, which give very clear descriptions of technical details appertaining to the administration of oxygen by catheter or by the tent method. These details of technique form a valuable addition from the clinical point of view.

**Chronic Miliary Tuberculosis.** By C. HOYLE, M.D., M.R.C.P. and M. VAIZEY, M.B., M.R.C.P. Pp. ix., 140. Illustrated. London : Oxford University Press (Humphrey Milford). 1937. Price 12s. 6d.—This small volume gives a lucid and most detailed account of a condition which must be described as relatively rare. The authors rather shrewdly remark that they hope to show that it is common enough to be important in the diagnosis of obscure and prolonged illnesses. If one takes an extreme case of chronic miliary tuberculosis and compares it with an extreme case of acute miliary tuberculosis the differentiation is simple and even obvious, but the dividing line is so broad and indistinct that there is a considerable number of cases which might quite fairly be classed either as rather long-drawn-out acute cases or alternatively rather rapid chronic cases. One point, however, is made quite clear, namely, that it is possible and actually not uncommon to have a case of chronic (or even acute) miliary tuberculosis of the lungs completely clearing up and a few years afterwards showing no abnormal physical signs in the chest, either by physical or X-ray examination.

**The Control of Tuberculosis in England, Past and Present.** By G. G. KAYNE, M.D., M.R.C.P., D.P.H. Pp. xiv., 188. London : Oxford University Press (Humphrey Milford). 1937. Price 8s. 6d.—This interesting little book could be read with profit by general practitioners and even general readers as well as by administrators and tuberculosis officers, for whom it is primarily intended. It is divided into three parts—the first two being a very lucid account of the administrative measures that have been taken in England for the control of tuberculosis, while the third part examines the problem as it exists to-day. This includes a detailed account of the average tuberculosis scheme with references to all the Ministry orders, etc., and inevitably ends with a forecast of future developments. These, Dr. Kayne is convinced, can only result in a State Medical Service. Even those who object to a State Service on principle must admit that Dr. Kayne puts his case clearly and reasonably. In fact, his attitude appears to be that, while not particularly anxious for a State Service, he can see no alternative likely to give a comparable efficiency.

**Iodine Metabolism and Thyroid Function.** By A. W. ELMER, M.D. Pp. xvii., 605. Illustrated. London: Oxford University Press (Humphrey Milford). 1938. Price 30s.—Although this book bears the publisher's imprint of the Oxford University Press, the sheets were printed in Poland. The paper is of the poorest quality and the setting of the type is unfamiliar, particularly the paragraph-headings, which are made striking by wide spacing of letters of the same size and from the same fount as are used in the body of the text. The author is to be congratulated on his translator, whose English is smooth and elegant, although occasionally through ignorance of current medical terms his English requires a little thought; as, for instance, "navel blood," which is a distinctly unfamiliar expression to use for blood taken from the umbilical cord at the time of parturition: "placental" would probably be better understood. "Undirectly" for "indirectly" (page 333) is perhaps only a printer's error. But on the same page we find that the presence of thyroxine could not be "stated": surely the proper translation is "proved" or "verified." On page 334 there is an odd sentence beginning: "It is not excluded that the difference in size of the cortex is due to anything more than . . . "; the true English appears to be: "It is possible that the difference in size . . . is only due to. . . ." The large section of the work devoted to the Physiology of Iodine Metabolism is fascinating reading. The conspectus of the literature is most valuable and the author's own observations are interesting and well-placed. In dealing with the Pathology of Iodine Metabolism the author contents himself with considering thyrotoxicosis, hypothyreosis and simple goitre. Here again the work done by the author himself and by others is admirably marshalled. Dr. Elmer is most critical in the acceptance of blood and urine analyses as evidence of normal or abnormal Iodine Metabolism, and on page 524 describes his own method of carrying out an "iodine tolerance test," which he claims shows a striking difference between cases with "euthyroidism" and those with thyrotoxicosis, non-toxic goitre and hypothyroidism. This is a book well worth reading; it is indispensable for a full realization of our present knowledge of thyroid function and malfunction.

**The Health of the Nation and Deficiency Diseases.** By J. MABERLY, M.R.C.S., L.R.C.P. Pp. xii., 118. London: Baillière, Tindall & Cox. 1938. Price 5s.—In this little book the author gives a short account of the vitamins and the deficiency diseases and a long dissertation on our present methods of milling wheat, methods which he claims are causing disease and death among the people of the East and are slowly undermining the physique and vigour of a large section of the people of England. One chapter is devoted to describing how certain patients suffering from acute poliomyelitis recovered their health under the author's care after the administration of iodized tincture of guaiacol. He has used this drug in various bacterial infections with advantage, as it has seemed to him, to the patients. Pellagra, the anæmias, and diabetes mellitus also come under consideration. The book as a whole is of no particular interest.

**Psyche and the Physiologists.** By E. G. D. DRURY, M.D., B.S., D.P.H. Pp. viii., 98. London: H. K. Lewis & Co. Ltd. 1938. Price 5s.—He is a brave man who attempts to invade that no-man's land which lies between physiology and psychology. Both camps probably regard him as an alien, and his very friendliness with the opposite side enhances this suspicion. The prospective reader can be reassured in the case of this book, as the outstanding characteristics are the author's wide knowledge of both specialities, combined with marked intellectual honesty. Here is an author who has combined wide reading with general practice and has culled much wisdom from both. All theories have obviously been applied to the touch-stone of experience. Particularly interesting is the author's review of recent work on acetyl-choline and its relation to clinical medicine. The book shows wide knowledge of contemporary medical literature and is written in a style that should make a wide appeal both to general practitioners and research workers. The chapter entitled "Labels and Luggage" will appeal to all interested in the medical aspects of education and the whole book is sufficiently short to form a very pleasant evening's reading.

**Alcohol and Human Life.** Second Edition. By C. C. WEEKS. Pp. xii., 455. Illustrated. London: H. K. Lewis & Co. Ltd. 1938. Price 6s.—This is partly a revision of *Alcohol and the Human Body* by the late Sir Victor Horsley and Dr. Mary Sturge. The second edition comes at an opportune moment in view of the temporary increase in the consumption of alcohol in this country. To all who are speaking or teaching on the subject of alcohol this book will prove a veritable store-house of facts and figures, set forth by the author in the broadest and most dispassionate manner. When it is realized that Great Britain is spending annually more than two hundred and fifty millions on "this drug" it is obvious that all medical practitioners and students should be made fully acquainted with the scientific aspects of the alcohol question. Of especial interest at the present time is the reference to the use of cocktails, and the chapter devoted to alcohol in relation to motor driving and road traffic accidents. In the latter the technique of the quantitative tests for alcohol in the blood is described, and their value discussed. The author is opposed to the compulsory use of such a test, but is of opinion that if invoked voluntarily, it might be of great value in clearing accused persons of a charge when they know they have only consumed a trifling amount of alcohol.

**Handbook of Sanitary Law.** Twelfth Edition. By B. BURNETT HAM, M.D., D.P.H. Pp. xxii., 355. London: H. K. Lewis & Co. Ltd. 1938. Price 7s. 6d.—The mass of recent public health legislation has necessitated that this handbook be almost completely re-written. The book has now been brought completely up to date, and includes such recent legislation as the Factories Act, 1937, and the Food and Drugs Act of 1938. In fact, the law relating to public health administration has been clearly and concisely stated without the burden of intricate legal phraseology, which is always cumbersome to the student. The handbook will be an invaluable aid to the student studying from the larger text-books on the subject, which are frequently not quite up to date on the question of legal obligations and requirements. It will have particular appeal to students studying for the Diploma of Public Health, but it is rather advanced for the undergraduate.

**Modern Anæsthetic Practice.** Edited by Sir HUMPHRY ROLLESTON, Bart., G.C.V.O., K.C.B., M.D., F.R.C.P., and A. A. MONCRIEFF, M.D., F.R.C.P. Pp. 231. London: Eyre & Spottiswoode Ltd. 1938. Price 10s. 6d.—In this book the subject is divided into twelve sections, each of which is dealt with by an author who has special knowledge of that particular branch of anæsthesia, the result being that the matter contained is practical and up to date. In the introduction the reader is warned that the complicated nature of modern methods and apparatus may be a source of danger unless handled with the necessary care and attention to detail. The chapter on nitrous oxide includes a very useful account of the significance of cyanosis with this reagent, pointing out that in exsanguinated subjects death may easily occur without any premonitory blueness ; and offering very clear guidance as to which type of patient may be expected to be suitable for nitrous oxide anæsthesia and which not. In the section on spinal anæsthesia a special point is made of the risk that this procedure may mean for patients who have suffered from severe hæmorrhage or for those who from any cause are *in extremis.* The author draws attention to the fact that certain of the complications sometimes occurring with this form of anæsthesia have yielded to modern methods of treatment, as, for instance, retention of urine, which generally can be overcome quickly by administering a drug of the acetyl-choline group such as doryl. Every section has been dealt with in the same clear style, and the authors are to be congratulated on having presented the profession with a book which is full of useful information, and which cannot fail to be appreciated. The two chapters which will probably prove of especial value to the general practitioner are those which deal respectively with post-operative care and with anæsthesia in children. The book is nicely produced, and has been furnished with a good index.

**Imperial and Metric Conversion Scales.** By J. W. THORNTON, M.A., B.M., M.R.C.P. Bristol: John Wright & Sons, Ltd. 1938. Price 6s.—For those to whom the manipulation of figures is a painful process, to be avoided whenever possible, but who, nevertheless, must use both the Metric and the Imperial systems of Weights and Measures, some means of comparing the two systems becomes a necessity. The Conversion Scales worked out by Dr. Thornton meet this need with the maximum convenience and scope. They cover the range between 100 kilograms and 1 milligram, and between 100 mils and 1 mil. This means between 220 lbs. (15 stone 10 lbs.) and 1/64th of a grain. The range can be extended on either side indefinitely by the use of multiples of 10. The scales can be used, easily and accurately, without the user being aware of the fact that they are logarithmic scales, or knowing what is, exactly, a logarithmic scale. What becomes evident is the great convenience of this method of comparing quantities. The Metric measurements take their calculable positions on the left of a line, and the erratic Imperial measurements on the right. The quantities are brought into close comparison in this way, and can be read off immediately.

**Fifty Years of Bayer Remedies, 1888-1938.** Bayer Products Ltd.—This very attractive brochure issued by the house of Bayer must be of considerable interest to the profession of medicine in that it is in itself a history of synthetic chemicals for the period. Its illustrations indicate, too, the predominance of pre-war Germany in this field. Phenacetin, antipyrin and aspirin are perhaps outstanding examples of Bayer's earlier products; while Ehrlich's years of painstaking experiments culminated in salvarsan and neo-salvarsan, the essentials of the V.D. clinics. In Bayer's aspirin, sold originally in 1 oz. bottles at 1s. per oz., we hardly foresaw the sale of one million tablets per year in Bristol alone. Novocaine was, perhaps, their leading contribution to infiltration anæsthesia, prominal and evipan their most important additions to the barbiturates; plasmoquine their main contribution to the treatment of malaria. Pioneers in the field of sulphonamides, prontosil has been possibly their greatest modern contribution to chemo-therapy. The book well represents fifty years of unceasing research, and its arrangement in sections facilitates reading.

